# Individual Preferences for Influenza Vaccine in Iran: A Discrete Choice Experiment

**DOI:** 10.1002/puh2.70458

**Published:** 2026-11-13

**Authors:** Sanaz Parvizi, Mohsen Mehrara, Ali Taiebnia

**Affiliations:** ^1^ Faculty of Economics University of Tehran Tehran Iran

**Keywords:** discrete choice experiment, influenza vaccine, Iran, vaccine preference

## Abstract

**Background and Aims:**

Influenza vaccination is a key preventive measure against seasonal influenza. Understanding public preferences for influenza vaccine attributes is essential for designing effective vaccination strategies. This study examined individuals’ preferences for influenza vaccine attributes in Iran and identified factors associated with vaccine choice.

**Methods:**

A discrete choice experiment (DCE) was conducted among 226 adults in Tehran province between September and December 2024. Seven vaccine attributes were evaluated: effectiveness, risk of severe side effects, risk of mild side effects, number of doses, duration of protection, location of manufacture, and price. Conditional and mixed logit models were used to estimate participants’ preferences, and model performance was assessed using the log‐likelihood, Akaike information criterion (AIC), and Bayesian information criterion (BIC).

**Results:**

Vaccines with higher effectiveness were more likely to be selected, whereas higher risks of severe side effects and higher prices were associated with a lower likelihood of vaccine selection. The risk of mild side effects, duration of protection, and location of manufacture showed limited or no association with vaccine choice in the conditional logit model, although the mixed logit model suggested a lower preference for domestically manufactured vaccines. Preference heterogeneity was observed, with age, marital status, education, and health insurance status being associated with differences in preferences for specific vaccine attributes.

**Conclusion:**

Vaccine effectiveness, safety, and affordability appeared to be the most important attributes associated with influenza vaccine choice in this study. These findings suggest that public health strategies emphasizing vaccine effectiveness and safety while improving affordability may help increase influenza vaccine uptake in Iran.

## Graphical abstract

A discrete choice experiment among 226 adults in Tehran province examined preferences for key influenza vaccine attributes. Vaccine effectiveness, risk of severe side effects, price, and dosing schedule were associated with vaccine choice. Higher effectiveness, lower risks of severe side effects, and lower prices were preferred, whereas participants also showed a preference for a two‐dose schedule. Preference heterogeneity was observed across demographic and socioeconomic characteristics. These findings may help inform influenza vaccination strategies that consider effectiveness, safety, and affordability in Iran.

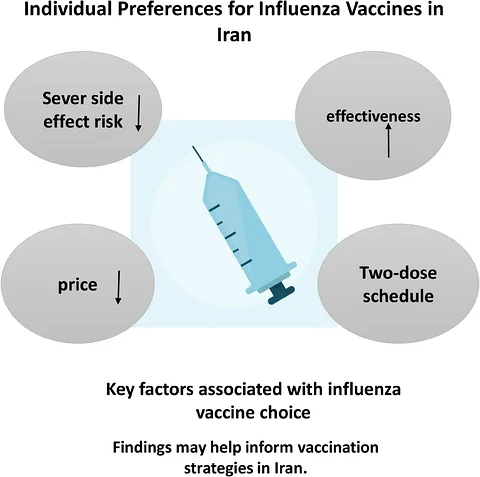

## 1 Introduction

Influenza is a highly contagious viral respiratory infection that affects millions of people worldwide each year and remains a major public health concern [1]. Although most infections are self‐limiting, influenza can lead to severe complications, particularly among young children, older adults, pregnant women, and individuals with chronic medical conditions. Consequently, the World Health Organization (WHO) recommends annual influenza vaccination for all individuals aged 6 months and older, with priority given to populations at increased risk of severe disease and those in close contact with high‐risk individuals [2].

Seasonal influenza vaccination is the most effective strategy for preventing influenza and its associated complications [3]. However, vaccine effectiveness varies according to several factors, including the match between vaccine strains and circulating viruses, vaccine formulation, and recipient characteristics, such as age and health status [4]. Despite the well‐established benefits of vaccination, influenza vaccine coverage in many countries remains below the targets recommended by the WHO, particularly among populations at increased risk of complications [5].

In Iran, influenza vaccines are available through both public and private healthcare providers. High‐risk groups may receive vaccination free of charge, whereas others typically obtain vaccines through private purchase. Both domestically manufactured and imported vaccines are available, with formulations based on WHO recommendations. Although vaccine availability has improved in recent years, vaccination coverage remains below recommended levels, highlighting the need to better understand the factors that influence vaccine‐related decision‐making.

Individuals' vaccination decisions are influenced by both personal characteristics (e.g., age, education, income, and health insurance status) and vaccine attributes (e.g., effectiveness, safety, price, dosing schedule, and duration of protection). Discrete choice experiments (DCEs) provide a well‐established approach for quantifying the relative importance of these attributes and examining heterogeneity in individual preferences [6, 7, 8, 9, 10, 11]. Although DCEs have been widely used to investigate influenza vaccine preferences in several countries [6, 7, 8], evidence from Iran remains unavailable.

Therefore, this study aimed to (1) examine individuals' preferences for influenza vaccine attributes in Iran and (2) assess whether these preferences varied according to sociodemographic characteristics. The findings may provide evidence to inform vaccination policies, communication strategies, and immunization program planning aimed at improving influenza vaccine uptake in Iran.

## 2 Methods

This study employed a DCE to investigate individuals’ preferences for influenza vaccine attributes in Iran. DCE is a stated‐preference method that is widely used in health economics and public health research to quantify the relative importance of product or service attributes and to examine the trade‐offs individuals make when choosing between hypothetical alternatives [6, 7, 8, 9, 10, 11].

Participants completed a series of hypothetical choice tasks designed to simulate real‐world vaccination decisions [12]. In each choice task, respondents selected their preferred option from two hypothetical influenza vaccines and a no‐vaccination alternative. The vaccine alternatives differed according to seven attributes, including vaccine effectiveness, risk of severe side effects, risk of mild side effects, number of doses, duration of protection, location of manufacture, and price [13, 14, 15]. By systematically varying these attributes across choice tasks, the DCE enabled estimation of the relative importance of each attribute and the trade‐offs participants were willing to make when selecting between vaccination alternatives [6, 7, 8, 16].

This approach offers several advantages over conventional survey methods. It allows multiple vaccine attributes to be evaluated simultaneously, quantifies the relative importance of each attribute, and captures heterogeneity in individuals’ preferences, thereby providing evidence that can inform vaccination policy and public health decision‐making [6, 16].

### 2.1 Attributes and Their Levels

The vaccine attributes and their corresponding levels were identified through a comprehensive review of the published literature on DCEs evaluating vaccine preferences [6, 7, 8, 11, 13, 15, 17] and refined through consultation with vaccine experts. The preliminary set of attributes was subsequently evaluated in a pilot study to assess its clarity, relevance, and comprehensibility. On the basis of these findings, seven attributes were selected for inclusion in the final DCE. The selected attributes and their levels are presented in Table 1.

- Vaccine effectiveness: Four effectiveness levels (20%, 40%, 60%, and 80%) were included to represent the expected level of protection against influenza infection.
- Risk of severe side effects: This attribute was defined at three levels (1, 10, and 100 per 1,000,000 vaccinated individuals) to represent the probability of severe adverse events, such as anaphylaxis.
- Risk of mild side effects: Three levels (1/10, 3/10, and 5/10) were included to represent the probability of experiencing mild adverse reactions, such as injection‐site soreness or fever.
- Number of doses: This attribute comprised three levels: one dose, two doses, and more than two doses.
- Duration of protection: Protection duration was presented at three levels: 3, 6, and 12 months.
- Location of manufacture: Two levels were included: domestically manufactured and imported vaccines.
- Price: Four price levels were considered: free of charge, 2,000,000, 4,000,000, and 6,000,000 IRR.

**TABLE 1:** Attributes and levels included in the discrete choice experiment.

Attributes	Effectiveness (%)	Risk of severe side effects	Risk of mild side effects	Number of doses	Duration of protection (months)	Location of manufacture	Price (IIR)
Levels	20	1/1,000,000	1/10	One dose	3	Imported vaccine	Free
	40	10/1,000,000	3/10	Two doses	6	Domestic vaccine	2,000,000
	60	100/1,000,000	5/10	More than two doses	12		4,000,000
	80						6,000,000

The complete list of attributes and their corresponding levels is presented in Table 1.

### 2.2 DCE Design and Questionnaire

Given the large number of possible hypothetical influenza vaccine profiles generated by the seven attributes and their corresponding levels (4^2^ × 3^4^ × 2 = 2592), presenting all possible profiles to respondents would have been impractical and cognitively burdensome. Therefore, a *D*‐efficient partial‐profile design was used to generate a manageable set of hypothetical choice tasks while maintaining statistical efficiency [6, 7, 8, 13, 15, 17, 18, 19, 20].

The final experimental design comprised 36 choice tasks, which were divided into three blocks of 12 choice tasks. Each participant completed one block consisting of 12 choice tasks. In each task, respondents were asked to choose their preferred option from two hypothetical influenza vaccine alternatives and a no‐vaccination alternative. An example of a discrete choice task is provided in Table S1.

The questionnaire consisted of three sections. The first section collected participants’ sociodemographic characteristics, including age, sex, marital status, educational level, occupation, household income, and health insurance status. The second section included the DCE, in which participants completed the assigned choice tasks. Before completing the DCE, participants were provided with clear instructions explaining the study objectives, vaccine attributes, and attribute levels to ensure a consistent understanding of the hypothetical scenarios. The third section collected information on participants' influenza vaccination history and other relevant health‐related characteristics [6, 7, 8, 21].

### 2.3 Collect Data and Sample Size

Data were collected through face‐to‐face interviews conducted between September and December 2024 in Tehran province, Iran. Participants were recruited using a convenience sampling approach from public health centers, community settings, and educational institutions. Individuals were eligible to participate if they were 18 years of age or older, were residents of Tehran province, and were able to provide informed consent. Individuals who were unable to complete the questionnaire because of language barriers or cognitive impairment were excluded.

To minimize interviewer selection bias, trained interviewers systematically approached potential participants at each recruitment site. Although the sample was not randomly selected from the general population, efforts were made to recruit participants with diverse sociodemographic characteristics, including age, sex, educational attainment, and income level, to improve the diversity of the study sample.

The sample size was determined according to the recommendations of Johnson and Orme using the following formula: *N* > 500*c*/(*t* × *a*), where *c* represents the largest number of levels among all attributes, *t* is the number of choice tasks, and *a* is the number of alternatives per choice task [6, 7, 11, 19, 20, 22]. On the basis of this calculation, the minimum required sample size was 56 participants. To increase statistical power and facilitate subgroup analyses, a total of 226 participants were recruited.

### 2.4 Statistical Analyses

A DCE was conducted to investigate participants' preferences for influenza vaccine attributes. The dependent variable was the vaccination option selected in each choice task, whereas the independent variables were the levels of the seven vaccine attributes.

A conditional logit model was first estimated to examine participants' preferences. This model assumes homogeneous preferences across individuals and that the independence of irrelevant alternatives (IIA) assumption holds [11, 19]. Owing to its simplicity and ease of interpretation, the conditional logit model is commonly used as a baseline model in DCE studies. However, because individuals may differ in their preferences, a mixed logit model was also estimated. The mixed logit model allows for random preference heterogeneity, accounts for correlation among repeated choices made by the same respondent, and relaxes the IIA assumption [6, 7, 8, 11, 12, 19, 20]. Consequently, it provides greater flexibility in modeling individual preferences and is widely recommended for analyzing DCE data in health economics.

The utility function was specified as follows:

*U_ij_* = *β*_0_ + *β*_1_ Effectiveness40*_ij_* + *β*_2_ Effectiveness60*_ij_* + *β*_3_ Effectiveness80*_ij_* + *β*_4_ Risk severe side effect10*_ij_* + *β*_5_ Risk severe side effect100*_ij_* + *β*_6_ Risk mild side effect3*_ij_* + *β*_7_ Riskmildsideeffect5*_ij_* + *β*_8_ Ndoses2*_ij_* + *β*_9_ morthan2*_ij_* + *β*_10_DurationProtection6*_ij_* + *β*_11_ Duration Protection 12*_ij_* + *β*_12_ Location Manufacture Domestic*_ij_* + *β*_13_Price*_ij_*.

In the regression analysis, the estimated coefficients represented the association between each vaccine attribute and the likelihood of selecting a vaccination alternative. The model included a constant term (*β*_0_), representing an individual's general preference for vaccination relative to no vaccination, as well as coefficients for each vaccine attribute [6, 18, 19, 20]. Dummy variables were used for all categorical attributes, whereas price was modeled as a continuous variable [6, 11, 18, 19].

Model performance was assessed using the log‐likelihood, Akaike information criterion (AIC), and Bayesian information criterion (BIC), with lower values indicating a better model fit. Participants’ characteristics were summarized using frequencies and percentages. A two‐sided *p* value < 0.05 was considered statistically significant. All statistical analyses were performed using Stata version 16.

## 3 Results

### 3.1 Individuals’ Characteristics

Table 2 summarizes the sociodemographic characteristics of the study participants. The study sample comprised predominantly female participants (68%), whereas males accounted for 32% of the sample.

**TABLE 2:** Individuals’ characteristics.

	*N*	%
**Gender**		
Male	72	32
Female	154	68
**Age**		
18–29	132	58.4
30–39	52	23
40–49	34	15
50–59	6	2.6
≥60	2	1
**Education**		
≤Diploma	0	0
Diploma	64	28
Bachelor	128	57
Master	28	12
PhD	6	3
**Income monthly**		
≤50,0000,000	18	8
50,000,000–100,000,000	108	48
≥100,000,000	100	44
**Job**		
Employed	88	39
Others (students, retired)	138	61
**Marital status**		
Married	100	44
Single	126	56
**Child**		
Yes	86	38
No	140	62
**Health insurance**		
Yes	178	79
No	48	21

In terms of age, participants aged 18–29 years represented the largest group (58.4%), followed by those aged 30–39 years (23%) and 40–49 years (15%). Participants aged 50–59 years and ≥60 years accounted for 2.6% and 1%, respectively.

Regarding educational attainment, 57% of participants held a bachelor's degree, 28% had a diploma, 12% had a master's degree, and 3% had a PhD.

With respect to monthly income, 8% of participants reported a monthly income of ≤50,000,000 IRR, 48% reported an income between 50,000,000 and 100,000,000 IRR, and 44% reported an income of ≥100,000,000 IRR (exchange rate: $1 = 502,510 IRR).

Overall, 39% of participants were employed, whereas 61% were classified as others (including students and retired individuals). In addition, 44% of participants were married, and 56% were single. Regarding family status, 38% of participants had at least one child, whereas 62% had no children. Most participants (79%) had health insurance, whereas 21% did not.

### 3.2 Estimation of Preferences for Influenza Vaccine

We first estimated a conditional logit model to examine participants’ preferences for influenza vaccine attributes. The model showed a log‐likelihood of −7356.771, an AIC of 14,741.54, a BIC of 14,837.05, and a McFadden's pseudo‐*R* ^2^ of 0.0121. The results are presented in Table 3.

**TABLE 3:** (A) Summary of model fit statistics and (B) conditional logit model estimates of preferences for influenza vaccine attributes.

(A)							
Model	*N*	LL (null)	LL (model)	*df*	AIC	BIC	McFadden's pseudo‐R^2^
**Fit statistics**	6780	−7446.685	−7356.771	14	14,741.54	14,837.05	0.0121

(B)					
Vaccine attribute	Coefficients	Standard error	*z*	*p* value	95% Confidence interval
Vaccine effectiveness (40%)	0.122	0.058	2.090	0.03	0.008, 0.236
Vaccine effectiveness (60%)	0.210	0.060	3.520	<0.001	0.093, 0.327
Vaccine effectiveness (80%)	0.416	0.066	6.28	<0.001	0.286, 0.546
Mild side effect (3/10)	−0.019	0.052	−0.36	0.71	−0.122, 0.084
Mild side effect (5/10)	−0.132	0.056	−2.36	0.01	−0.241, −0.022
Severe side effect (10/1,000,000)	−0.165	0.052	−3.17	0.002	−0.267, −0.063
Severe side effect (100 per 1,000,000)	−0.330	0.051	−6.49	<0.001	−0.430, −0.230
Two doses	0.130	0.058	2.25	0.02	0.017, 0.243
More than two doses	0.053	0.049	1.07	0.28	−0.044, 0.149
Duration of protection (6 months)	0.051	0.052	0.97	0.33	−0.052, 0.153
Duration of protection (12 months)	−0.101	0.070	−1.44	0.15	−0.239, 0.037
Price	−0.044	0.019	−2.26	0.02	−0.082, −0.006
Domestic manufacture	−0.065	0.040	−1.63	0.10	−0.144, 0.013
No vaccination	−0.044	0.083	−0.53	0.59	−0.206, 0.119

*Note:* **Reference categories**: vaccine effectiveness (20%); mild side effects (1/10); severe side effects (1 per 1,000,000); one‐dose schedule; duration of protection (3 months); and imported vaccine.

Abbreviations: AIC, Akaike information criterion; BIC, Bayesian information criterion.

Compared with the reference level of 20% vaccine effectiveness, participants were more likely to select vaccines with 60% effectiveness (*β* = 0.210, *p* < 0.001) and 80% effectiveness (*β* = 0.416, *p* < 0.001), indicating a clear preference for vaccines offering higher levels of effectiveness.

Safety was also an important consideration in participants’ choices. Vaccines with a greater risk of severe side effects were less likely to be selected. Compared with the reference category, both the 10 per 1,000,000 (*β* = −0.165, *p* = 0.002) and 100 per 1,000,000 (*β* = −0.330, *p* < 0.001) severe side‐effect levels were associated with lower preference. For mild side effects, only the 5/10 level showed evidence of a lower preference (*β* = −0.132, *p* = 0.01), whereas the 3/10 level was not statistically significant.

With regard to the number of doses, participants showed a preference for a two‐dose schedule over a single‐dose schedule (*β* = 0.130, *p* = 0.02). In contrast, requiring more than two doses did not appear to affect vaccine choice (*p* = 0.28).

No statistically significant preference was observed for either 6 or 12 months of protection, suggesting that the duration of protection was not an important attribute in participants’ vaccine choices.

As expected, higher vaccine prices were associated with a lower probability of vaccine selection (*β* = −0.044, *p* = 0.02).

Finally, neither the location of manufacture (*β* = −0.065, *p* = 0.10) nor the non‐vaccination alternative (*β* = −0.044, *p* = 0.59) showed evidence of a statistically significant effect on participants’ choices.

To account for unobserved preference heterogeneity, we also estimated a mixed logit model (Table 4). The model achieved a log‐likelihood of −7347.38, an AIC of 14,748.76, a BIC of 14,932.94, and a McFadden's pseudo‐*R* ^2^ of 0.0133. Compared with the conditional logit model (log‐likelihood = −7356.77; AIC = 14,741.54; BIC = 14,837.05; and McFadden's pseudo‐*R* ^2^ = 0.0121), the mixed logit model yielded a higher log‐likelihood and a slightly higher McFadden's pseudo‐*R* ^2^, but higher AIC and BIC values after accounting for the larger number of estimated parameters. Thus, the information criteria did not provide evidence of an overall improvement in model fit for the mixed logit specification. Nevertheless, the mixed logit model was estimated to account for unobserved preference heterogeneity and to examine potential variation in individual preferences across respondents.

**TABLE 4:** (A) Summary of model fit statistics and (B) mixed logit model estimates of preferences for influenza vaccine attributes.

(A)							
Model	*N*	LL (null)	LL (model)	*df*	AIC	BIC	McFadden's pseudo‐R^2^
**Fit statistics**	6780	−7446.685	−7347.3782	27	14,748.76	14,932.94	0.0133

(B)						
Vaccine attributes	Coefficient	Standard error	*z*	*p* value	95% Confidence interval	Standard deviation
Vaccine effectiveness (40%)	0.049	0.106	0.460	0.64	−0.159, 0.256	1.057
Vaccine effectiveness (60%)	0.261	0.085	3.090	0.002	0.095, 0.427	0.099*
Vaccine effectiveness (80%)	0.535	0.116	4.630	<0.001	0.309, 0.762	0.808
Mild side effects (3/10)	−0.092	0.088	−1.040	0.29	−0.265, 0.081	0.365
Mild side effect (5/10)	−0.217	0.083	−2.610	0.009	−0.381, −0.054	0.086
Severe side effect (10 per 1,000,000)	−0.288	0.090	−3.200	0.001	−0.465, −0.111	0.00000188
Severe side effect (100 per 1,000,000)	−0.440	0.082	−5.360	<0.001	−0.601, −0.279	0.027
Two doses	0.080	0.099	0.800	0.42	−0.115, 0.274	1.143
More than two doses	−0.008	0.083	−0.100	0.92	−0.171, 0.155	0.966
Duration protection (6 months)	0.052	0.081	0.640	0.52	−0.106, 0.210	0.823
Duration of protection (12 months)	−0.086	0.102	−0.840	0.40	−0.286, 0.114	0.015
Price	−0.075	0.030	−2.480	0.01	−0.134, −0.016	0.315**
Domestic manufacture	−0.159	0.077	−2.070	0.03	−0.309, −0.008	0.880**
No vaccination	−0.282	0.148	−1.910	0.05	−0.572, 0.008	

*Note:* **Reference categories**: vaccine effectiveness (20%); mild side effects (1/10); severe side effects (1 per 1,000,000); one‐dose schedule; duration of protection (3 months); and imported vaccine.

Abbreviations: AIC, Akaike information criterion; BIC, Bayesian information criterion.

*p < 0.10.

**p < 0.05.

Compared with the reference level of 20% vaccine effectiveness, participants were more likely to select vaccines with 60% effectiveness (*β* = 0.261, *p* = 0.002) and 80% effectiveness (*β* = 0.535, *p* < 0.001).

Participants also showed lower preference for vaccines with higher risks of side effects. For mild side effects, only the 5/10 level was associated with a lower probability of vaccine selection (*β* = −0.217, *p* = 0.009), whereas the 3/10 level did not show a statistically significant difference. Similarly, vaccines with severe side‐effect risks of 10 per 1,000,000 (*β* = −0.288, *p* = 0.001) and 100 per 1,000,000 (*β* = −0.440, *p* < 0.001) were less likely to be selected than the reference category.

No statistically significant preference was observed for either a two‐dose schedule (*β* = 0.080, *p* = 0.42) or more than two doses (*β* = −0.008, *p* = 0.92) compared with a single‐dose schedule.

Likewise, neither 6 months (*β* = 0.052, *p* = 0.52) nor 12 months (*β* = −0.086, *p* = 0.40) of protection showed evidence of influencing vaccine choice.

Higher vaccine prices were associated with a lower probability of vaccine selection (*β* = −0.075, *p* = 0.01).

Participants also showed lower preference for domestically manufactured vaccines than for imported vaccines (*β* = −0.159, *p* = 0.03).

Finally, the coefficient for the opt‐out (no vaccination) alternative was negative (*β* = −0.282, *p* = 0.05), suggesting that respondents generally preferred vaccination over the non‐vaccination option.

On the basis of the estimated standard deviations, significant preference heterogeneity was observed for several vaccine attributes, including 60% vaccine effectiveness, price, and place of manufacture, indicating that respondents differed in the importance they attached to these attributes.

To further explore the sources of this heterogeneity, interaction terms between sociodemographic characteristics and selected vaccine attributes were incorporated into the mixed logit model. As presented in Table 5, age, marital status, education, and health insurance status were associated with preferences for specific vaccine attributes.

**TABLE 5:** Mixed logit model with interaction terms between sociodemographic characteristics and selected vaccine attributes.

Vaccine attribute	Coefficient	Standard error	*z*	*p* value	95% confidence interval
No vaccination	−0.415	0.155	−2.68	0.007	−0.719, −0.111
Gender × vaccine effectiveness (60%)	0.146	0.105	1.39	0.16	−0.060, 0.352
Age × vaccine effectiveness (60%)	0.035	0.080	0.44	0.66	−0.121, 0.191
Education × vaccine effectiveness (60%)	0.030	0.069	0.43	0.68	−0.106, 0.165
Working × vaccine effectiveness (60%)	0.057	0.100	0.57	0.57	−0.139, 0.253
Marital status × vaccine effectiveness (60%)	−0.562	0.196	−2.87	0.004	−0.946, −0.179
Child × vaccine effectiveness (60%)	0.283	0.205	1.38	0.17	−0.118, 0.684
Income monthly × vaccine effectiveness (60%)	0.068	0.073	0.93	0.35	−0.075, 0.211
Health insurance × vaccine effectiveness (60%)	0.012	0.110	0.11	0.91	−0.202, 0.227
Gender × price	−0.062	0.030	−2.05	0.04	−0.122, −0.003
Age × price	0.066	0.025	2.64	0.008	0.017, 0.114
Education × price	−0.087	0.023	−3.71	<0.001	−0.133, −0.041
Working × price	−0.055	0.030	−1.87	0.06	−0.114, 0.003
Marital status × price	0.028	0.050	0.56	0.58	−0.070, 0.126
Child × price	−0.078	0.058	−1.35	0.18	−0.191, 0.035
Income monthly × price	−0.034	0.022	−1.55	0.12	−0.077, 0.009
Health insurance × price	−0.289	0.057	−5.10	0.001	−0.400, −0.178
Gender × domestic manufacture	0.050	0.084	0.59	0.55	−0.114, 0.214
Age × domestic manufacture	−0.061	0.068	−0.90	0.37	−0.194, 0.072
Education × domestic manufacture	0.209	0.062	3.36	<0.001	0.087, 0.332
Working × domestic manufacture	−0.022	0.082	−0.27	0.79	−0.182, 0.138
Marital status × domestic manufacture	−0.382	0.146	−2.63	0.009	−0.668, −0.097
Child × domestic manufacture	0.337	0.165	2.05	0.04	0.015, 0.660
Income monthly × domestic manufacture	−0.089	0.060	−1.48	0.14	−0.206, 0.029
Health insurance × domestic manufacture	−0.465	0.093	−4.99	<0.001	−0.647, −0.282
Vaccine effectiveness (40%)	0.129	0.069	1.85	0.06	−0.007, 0.265
Vaccine effectiveness (60%)	−0.069	0.285	−0.24	0.81	−0.628, 0.490
Vaccine effectiveness (80%)	0.529	0.105	5.05	<0.001	0.324, 0.734
Mild side effect (3/10)	−0.169	0.099	−1.70	0.09	−0.364, 0.026
Mild side effect (5/10)	−0.269	0.084	−3.19	0.001	−0.434, −0.104
Severe side effect (10 per 1,000,000)	−0.293	0.086	−3.43	0.001	−0.461, −0.126
Severe side effect (100 per 1,000,000)	−0.487	0.083	−5.91	<0.001	−0.649, −0.326
Two doses	0.062	0.076	0.80	0.42	−0.088, 0.211
More than two doses	−0.017	0.063	−0.27	0.79	−0.141, 0.107
Duration of protection (6 months)	0.027	0.087	0.30	0.76	−0.145, 0.198
Duration of protection (12 months)	−0.119	0.086	−1.38	0.17	−0.289, 0.050
Price	0.457	0.099	4.60	<0.001	0.262, 0.651
Domestic manufacture	−0.025	0.230	−0.11	0.91	−0.476, 0.426

*Note:* **Reference categories**: vaccine effectiveness (20%); mild side effects (1/10); severe side effects (1 per 1,000,000); one‐dose schedule; duration of protection (3 months); and imported vaccine. Interaction terms represent the association between sociodemographic characteristics and selected vaccine attributes.

A negative interaction was observed between marital status and vaccines with 60% effectiveness (*β* = −0.562, *p* = 0.004), suggesting that married participants were less likely than unmarried participants to select vaccines with 60% effectiveness.

A positive interaction was identified between age and vaccine price (*β* = 0.066, *p* = 0.008), suggesting that older participants may have been more willing to choose higher priced vaccines. This finding may reflect differences in perceived vaccine value or perceived vulnerability to influenza.

Education showed a negative interaction with vaccine price (*β* = −0.087, *p* < 0.001), indicating that participants with higher educational attainment were less likely to select higher priced vaccines. This pattern may reflect greater attention to cost‐effectiveness when making vaccination decisions.

Similarly, health insurance status was negatively associated with vaccine price (*β* = −0.289, *p* = 0.001), suggesting that insured participants were less likely to choose higher priced vaccines than uninsured participants.

With respect to the place of manufacture, participants with higher educational attainment were more likely to prefer domestically manufactured vaccines (*β* = 0.209, *p* < 0.001), whereas married participants were less likely to select domestically manufactured vaccines (*β* = −0.382, *p* = 0.009).

Having children was positively associated with preferences for domestically manufactured vaccines (*β* = 0.337, *p* = 0.04), which may reflect greater concerns regarding vaccine safety or quality among parents. In contrast, participants with health insurance were less likely to prefer domestically manufactured vaccines than those without health insurance (*β* = −0.465, *p* < 0.001).

Overall, these findings suggest that sociodemographic characteristics may contribute to heterogeneity in stated preferences for influenza vaccine attributes, particularly with respect to vaccine effectiveness, price, and place of manufacture.

## 4 Discussion

The present study examined individuals’ stated preferences for influenza vaccine attributes using a DCE and explored heterogeneity in these preferences across participants. Overall, the findings suggest that vaccine effectiveness and safety were among the most influential attributes associated with participants’ vaccine preferences.

Consistent with previous studies, participants showed stronger preferences for vaccines with higher effectiveness levels [6, 7, 8]. These findings suggest that vaccine effectiveness is an important attribute considered during vaccination decision‐making. Communicating evidence on both vaccine efficacy and real‐world effectiveness may therefore contribute to improving vaccine acceptance and uptake.

The results also indicate that participants were less likely to choose vaccines with higher risks of severe side effects. This pattern is consistent with previous evidence showing that concerns about vaccine safety influence vaccination decisions. Clear and transparent communication regarding the safety profile of influenza vaccines may help improve public confidence and support vaccine acceptance.

In contrast, mild side effects were less influential in participants’ stated preferences. Only higher levels of mild side‐effect risk were associated with lower vaccine preference, whereas lower levels showed no statistically significant association. Further research is warranted to better understand which types and severities of mild side effects are most relevant to vaccination decisions.

The number of vaccine doses showed limited influence on participants’ preferences. Although a two‐dose schedule was preferred in the conditional logit model, this association was not observed in the mixed logit model. This finding suggests that the importance of the dosing schedule may vary across individuals.

Similarly, the duration of vaccine protection was not associated with participants’ stated preferences in either model. This finding differs from some previous studies and may reflect differences in study populations, vaccine contexts, or experimental design.

Vaccine price was associated with participants’ preferences, with lower priced vaccines being more likely to be selected. This finding is consistent with previous research highlighting affordability as an important consideration in vaccination decisions [6]. Policies that improve vaccine affordability, such as financial subsidies or other pricing strategies, may help increase vaccine uptake.

The location of vaccine manufacture was not associated with vaccine choice in the conditional logit model. However, the mixed logit model suggested that respondents were less likely to select domestically manufactured vaccines. This finding may reflect differences in perceptions of vaccine quality, safety, or confidence in domestic production. Strengthening quality assurance systems and communicating regulatory standards transparently may help improve public confidence in domestically manufactured vaccines.

The interaction analysis further suggested that sociodemographic characteristics were associated with heterogeneity in vaccine preferences. In particular, marital status, age, education, and health insurance status were associated with preferences for selected vaccine attributes, indicating that vaccination preferences may differ across population subgroups.

Overall, the findings are broadly consistent with previous studies reporting that vaccine effectiveness is one of the most important determinants of influenza vaccine preference [6, 7, 8]. Likewise, the observed association between vaccine price and stated preferences agrees with earlier studies demonstrating the importance of affordability in vaccination decisions [6].

However, our findings differ from those reported by Li and Gong [11], who observed that a longer duration of protection was associated with stronger vaccine preferences. In contrast, no such association was observed in the present study. These differences may reflect variations in study populations, survey design, or contextual factors.

Differences were also observed regarding the place of vaccine manufacture. Although Li and Gong (2022) reported a preference for domestically manufactured vaccines, our mixed logit results suggested lower preferences for domestic vaccines, whereas the conditional logit model showed no statistically significant association. These differences may reflect variation in public perceptions across settings and healthcare contexts.

The observed preference heterogeneity is also consistent with previous studies suggesting that sociodemographic characteristics contribute to differences in vaccine preferences [6, 7, 11].

This study contributes to the limited evidence on influenza vaccine preferences in low‐ and middle‐income countries and provides evidence that may help inform vaccination policy. In particular, strategies aimed at improving vaccine affordability, strengthening communication regarding vaccine effectiveness and safety, and increasing public confidence in domestic vaccine production may support influenza vaccination programs.

Several limitations should be acknowledged. First, as with all DCEs, the study relied on hypothetical scenarios, which may not fully reflect real‐world behavior (hypothetical bias). Second, the experiment included a limited number of vaccine attributes and did not consider other potentially relevant factors, such as healthcare provider recommendations, vaccine availability, or trust in healthcare institutions. Third, participants were recruited using convenience sampling in Tehran province, which may limit the generalizability of the findings to the broader Iranian population. Finally, voluntary participation may have introduced self‐selection bias, as individuals with stronger views about vaccination may have been more likely to participate. Future studies using nationally representative samples and revealed preference data would help validate these findings.

## 5 Conclusions

This study examined stated preferences for influenza vaccine attributes and explored factors associated with vaccine choice among adults in Tehran, Iran. The findings suggest that vaccine effectiveness, safety, and price were important attributes associated with participants’ stated preferences, whereas sociodemographic characteristics contributed to preference heterogeneity. These findings may help inform public health strategies aimed at improving influenza vaccine acceptance and uptake. In particular, policies that enhance vaccine affordability, strengthen communication about vaccine safety and effectiveness, and increase public confidence in vaccination may support higher vaccine uptake.

## Author Contributions

**Sanaz Parvizi**: conceptualization, formal analysis, investigation, methodology, validation, visualization, writing – original draft preparation, writing – review and editing. **Mohsen Mehrara**: conceptualization, formal analysis, investigation, methodology, validation, visualization. **Ali Taiebnia**: conceptualization, formal analysis, investigation, methodology, validation, visualization. All authors have read and approved the final version of the manuscript, and the corresponding author (Sanaz Parvizi) confirms that I had full access to all of the data in the study and takes complete responsibility for the integrity of the data and the accuracy of the data analysis.

## Funding

The authors have nothing to report.

## Ethics Statement

The authors have followed the ethical principles outlined in the 2013 revision of the Declaration of Helsinki, which is a widely recognized international standard for ethical conduct in medical research. Additionally, the participation of individuals in the survey is completely voluntary and based on their own choice, without any external pressure or influence.

## Conflicts of Interest

The authors declare no conflicts of interest.

## Transparency Statement

The lead author, Sanaz Parvizi, affirms that the manuscript is an honest, accurate, and transparent account of the study being reported; that no important aspects of the study have been omitted; and that any discrepancies from the study as planned (and, if relevant, registered) have been explained.

## Supporting information




**Supporting File 1**: puh270458‐sup‐0001‐SuppMat.docx

## Data Availability

The data that support the findings of this study are available from the corresponding author upon reasonable request.
