# Supplementary material for: Individual Preferences for Influenza Vaccine in Iran: A Discrete Choice Experiment
Source: Public Health Chall. 2026 Nov 13;5(4):e70458. doi: 10.1002/puh2.70458 (PMC13764547; doi:10.1002/puh2.70458)
Supplement: Supplementary file 1 [file PUH2-5-e70458-s001.docx]

**Supporting Information**

**Supplementary table S1.**

**Example of a discrete choice experiment task presented to participant**

| Q1 | Vaccine A | Vaccine B | No vaccine |
| --- | --- | --- | --- |
| Effectiveness | 60% | 80% | 0% |
| risk of severe side effects | 100 per 1000000 individuals | 10 per 1000000 individuals | 0 |
| risk of mild side effects | 5 per 10 individuals | 5per 10 individuals | 0 |
| Number of doses | More than 2 doses | 1doses | 0 |
| duration of protection | 12months | 6 months | 0 |
| location of manufacture | Domestic vaccine | Imported vaccine | 0 |
| Price | 4,000,000 IRR | 4,000,000 IRR | 0 |
| Which one do you prefer? |  |  |  |
